# Draft Genome Sequence of the Endophytic Strain *Rhodococcus kyotonensis* KB10, a Potential Biodegrading and Antibacterial Bacterium Isolated from *Arabidopsis thaliana*

**DOI:** 10.1128/genomeA.00636-16

**Published:** 2016-07-07

**Authors:** Chi Eun Hong, Sung Hee Jo, Haeyoung Jeong, Jeong Mee Park

**Affiliations:** a Molecular Biofarming Research Center, Korea Research Institute of Bioscience and Biotechnology, Yuseong-Gu, Daejeon, Republic of Korea; b Department of Biosystems and Bioengineering, University of Science and Technology, Yuseong-Gu, Daejeon, Republic of Korea; c Super-Bacteria Research Center, Korea Research Institute of Bioscience and Biotechnology, Yuseong-Gu, Daejeon, Republic of Korea

## Abstract

*Rhodococcus kyotonensis* KB10 is an endophytic bacterium isolated from *Arabidopsis thaliana*. The organism showed mild antibacterial activity against the phytopathogen *Pseudomonas syringae* pv. tomato DC3000. This study reports the genome sequence of *R. kyotonensis* KB10. This bacterium contains an ectoine biosynthesis gene cluster and has the potential to degrade nitroaromatic compounds. The identified bacterium may be a suitable biocontrol agent and degrader of environmental pollutants.

## GENOME ANNOUNCEMENT

*Rhodococcus* species are noted for their metabolic versatility, including degradation of chemically diverse xenobiotics and bioconversion (1, 2). Moreover, they produce indole-3-acetic acid, which is one of the most active auxins that stimulate plant growth (3). *Rhodococcus* species show antagonistic activity against human pathogens and the phytopathogens *Ceratocystis fimbriata* and *Pseudomonas syringae* pv. tomato DC3000 (1, 4, 5). Therefore, *Rhodococcus* may be an attractive genus for the bioremediation of environmental pollutants and for biocontrol in an agricultural setting (6). Endophytic bacteria are beneficial to their host plants in that they provide protection against phytopathogens and other stresses (7). In a previous study, we isolated the leaf-inhabiting endophytic bacterium KB10 from apoplastic fluid extracts from the uninfected upper leaves of *Arabidopsis thaliana* plants infected with *Pseudomonas syringae* pv. tomato DC3000; the bacterium was identified as *Rhodococcus kyotonensis* based on sequence analysis of the 16S rRNA gene (5). *R. kyotonensis* was originally isolated from soil samples in Kyoto city, Japan (8). Phylogenetic analysis using the neighbor-joining method revealed that it is closely related to *Rhodococcus yunnanensis*, a mesophilic actinobacterium (9, 10).

Genome sequencing was performed using the Illumina HiSeq 2500 platform at the National Instrument Center for Environmental Management at Seoul National University (Seoul, Republic of Korea). A total of 1,930,607,782 paired reads (151 cycles) were generated from a library, with an average insert size of approximately 433 bp, which was constructed using the TruSeq Nano LT DNA sample preparation version 2 kit. Adaptor sequence removal, quality trimming, error correction, *de novo* assembly, and scaffolding were all performed using the A5-miseq pipeline (11). The assembly contained 44 scaffolds from 1,467,368,630 reads (average length, 134.4 bp), with a G+C content of 65.2%. The total scaffold length, maximum scaffold length, and *N*_50_ were 5,472,710 bp, 923,597 bp, and 212,310 bp, respectively. Automatic genome annotation conducted using the RAST server (12) identified 5,152 coding sequences and 63 RNAs, 40% of which were assigned to subsystems.

Potential secondary metabolites were analyzed using analysis with antiSMASH (13) and RAST (12). *R. kyotonensis* KB10 comprised 15 gene clusters, including an ectoine biosynthesis gene cluster. Ectoine is a highly soluble molecule that provides osmoprotection in highly saline environments and has potential industrial applications (14). Furthermore, KB10 may be useful for bioremediation because it harbors genes encoding enzymes that degrade nitroaromatic compounds (15, 16). The information presented herein will enable further study of the genetic and functional characteristics of *R. kyotonensis* KB10 and its potential for biocontrol and biodegradation.

### Nucleotide sequence accession numbers.

The whole-genome shotgun project has been deposited at DDBJ/ENA/GenBank under the accession no. LVHI00000000. The version described in this paper is version LVHI00000000.1.

## References

[1] Gürtler V, Mayall BC, Seviour R 2004 Can whole genome analysis refine the taxonomy of the genus *Rhodococcus*? FEMS Microbiol Rev 28:377–403. doi:10.1016/j.femsre.2004.01.001. 15449609

[2] Van der Geize R, Dijkhuizen L 2004 Harnessing the catabolic diversity of rhodococci for environmental and biotechnological applications. Curr Opin Microbiol 7:255–261. doi:10.1016/j.mib.2004.04.001. 15196492

[3] Tsavkelova EA, Cherdyntseva TA, Netrusov AI 2005 Auxin production by bacteria associated with orchid roots. Microbiology 74:46–53. doi:10.1007/s11021-005-0027-6. 15835779

[4] Hong CE, Jeong H, Jo SH, Jeong JC, Kwon SY, An D, Park JM 2016 A leaf-inhabiting endophytic bacterium, *Rhodococcus* sp. KB6, enhances sweet potato resistance to black rot disease caused by *Ceratocystis fimbriata*. J Microbiol Biotechnol 26:488–492. doi:10.4014/jmb.1511.11039. 26767576

[5] Hong CE, Jo SH, Moon JY, Lee J-S, Kwon S-Y, Park JM 2015 Isolation of novel leaf-inhabiting endophytic bacteria in *Arabidopsis thaliana* and their antagonistic effects on phytophathogens. Plant Biotechnol Rep 9:451–458. doi:10.1007/s11816-015-0372-5.

[6] Treadway SL, Yanagimachi KS, Lankenau E, Lessard PA, Stephanopoulos G, Sinskey AJ 1999 Isolation and characterization of indene bioconversion genes from *Rhodococcus* strain I24. Appl Microbiol Biotechnol 51:786–793. doi:10.1007/s002530051463. 10422226

[7] Rosenblueth M, Martínez-Romero E 2006 Bacterial endophytes and their interactions with hosts. Mol Plant Microbe Interact 19:827–837. doi:10.1094/MPMI-19-0827. 16903349

[8] Li B, Furihata K, Ding LX, Yokota A 2007 *Rhodococcus kyotonensis* sp. nov., a novel actinomycete isolated from soil. Int J Syst Evol Microbiol 57:1956–1959. doi:10.1099/ijs.0.64770-0. 17766854

[9] Zhang YQ, Li WJ, Kroppenstedt RM, Kim CJ, Chen GZ, Park DJ, Xu LH, Jiang CL 2005 *Rhodococcus yunnanensis* sp. nov., a mesophilic actinobacterium isolated from forest soil. Int J Syst Evol Microbiol 55:1133–1137. doi:10.1099/ijs.0.63390-0. 15879245

[10] Saitou N, Nei M 1987 The neighbor-joining method: a new method for reconstructing phylogenetic trees. Mol Biol Evol 4:406–425. 3447015 10.1093/oxfordjournals.molbev.a040454

[11] Coil D, Jospin G, Darling AE 2015 A5-miseq: an updated pipeline to assemble microbial genomes from Illumina MiSeq data. Bioinformatics 31:587–589. doi:10.1093/bioinformatics/btu661. 25338718

[12] Aziz RK, Bartels D, Best AA, DeJongh M, Disz T, Edwards RA, Formsma K, Gerdes S, Glass EM, Kubal M, Meyer F, Olsen GJ, Olson R, Osterman AL, Overbeek RA, McNeil LK, Paarmann D, Paczian T, Parrello B, Pusch GD, Reich C, Stevens R, Vassieva O, Vonstein V, Wilke A, Zagnitko O 2008 The RAST server: Rapid Annotations using Subsystems Technology. BMC Genomics 9:75. doi:10.1186/1471-2164-9-75. 18261238 PMC2265698

[13] Weber T, Blin K, Duddela S, Krug D, Kim HU, Bruccoleri R, Lee SY, Fischbach MA, Müller R, Wohlleben W, Breitling R, Takano E, Medema MH 2015 antiSMASH 3.0–a comprehensive resource for the genome mining of biosynthetic gene clusters. Nucleic Acids Res 43:W237–W243. doi:10.1093/nar/gkv437. 25948579 PMC4489286

[14] Reshetnikov AS, Khmelenina VN, Mustakhimov II, Trotsenko YA 2011 Genes and enzymes of ectoine biosynthesis in halotolerant methanotrophs, p 15–30. *In* Amy CR, Stephen WR (), Methods in enzymology, vol 495. Academic Press, Cambridge, MA. 10.1016/B978-0-12-386905-0.00002-4 21419912

[15] Ghosh A, Khurana M, Chauhan A, Takeo M, Chakraborti AK, Jain RK 2010 Degradation of 4-nitrophenol, 2-choloro-4-nitrophenol, and 2,4-dinitrophenol by *Rhodococcus imtechensis* strain RKJ300. Environ Sci Technol 44:1069–1077. doi:10.1021/es9034123. 20050667

[16] Gemini VL, Gallego A, de Oliveira VM, Gomez CE, Manfio GP, Korol SE 2005 Biodegradation and detoxification of -nitrophenol by *Rhodococcus wratislaviensis*. Int Biodeterior Biodegradr 55:103–108. doi:10.1016/j.ibiod.2004.08.003.

